# The development and evaluation of a continuous flow process for the lipase-mediated oxidation of alkenes

**DOI:** 10.3762/bjoc.5.27

**Published:** 2009-06-02

**Authors:** Charlotte Wiles, Marcus J Hammond, Paul Watts

**Affiliations:** 1 Department of Chemistry, The University of Hull, Cottingham Road, Hull, HU6 7RX, UK; 2 Chemtrix BV, Burgemeester Lemmensstraat 358, 6163JT, Geleen, The Netherlands

**Keywords:** *Candida antarctica* lipase B, continuous flow, epoxidation, hydrogen peroxide, lipase, micro reactor, Novozym^®^ 435, peracids

## Abstract

We report the use of an immobilised form of *Candida antarctica* lipase B, Novozym^®^ 435, in a preliminary investigation into the development of a continuous flow reactor capable of performing the chemo-enzymatic oxidation of alkenes in high yield and purity, utilising the commercially available oxidant hydrogen peroxide (100 volumes). Initial investigations focussed on the lipase-mediated oxidation of 1-methylcyclohexene, with the optimised reaction conditions subsequently employed for the epoxidation of an array of aromatic and aliphatic alkenes in 97.6 to 99.5% yield and quantitative purity.

## Introduction

In addition to their synthetic value as intermediates in the preparation of diols, alcohols, hydroxyesters and alkenes, epoxides are a key raw material in many industrial processes, finding application in adhesives, polymers, coatings and paints [1–2], with some epoxides even exhibiting biological activity [3–4]. As such, over the years, convenient and efficient techniques have been sought for their preparation.

Within the research laboratory, epoxidation of alkenes is achieved using organic peroxides and metal catalysts [5] or peracids [6] such as *m*-chloroperbenzoic acid (*m*-CPBA) [7–8]. The hazardous nature of these techniques and the potential to hydrolyse the epoxide [9] however precludes their use on a large scale. Many of the epoxides produced industrially are synthesised using the chlorohydrin method, or *via* in situ generated peracids derived from formic acid or acetic acid (**1**)/hydrogen peroxide (**2**). As H_2_O_2_ (**2**) is itself not sufficiently electrophilic to epoxidise a non-conjugated double bond directly, its use in the formation of a peracid has afforded a route to the epoxidation of alkenes in the presence of a strong mineral acid [10–11]. Along with the numerous safety issues that these methods present, the generation of large quantities of chlorinated by-products and acidic waste make these approaches undesirable; not only from a disposal stand point, but also with respect to product quality, with reduced chemoselectivity observed in strongly acidic media.

As a means of addressing these problems, the past 20 years has seen a series of authors investigate the use of a chemo-enzymatic process for the oxidation of olefins, based on the enzymatic formation of peracids from carboxylic acids and oxidants such as H_2_O_2_ (**2**) and urea–hydrogen peroxide (UHP, **3**) [12].

For this transformation, the biocatalysts of choice are lipases (E.C. 3.1.1.3), which are a group of water soluble enzymes that catalyse the hydrolysis of lipid substrates, i.e. triglycerides and fats, in biological systems and are a subclass of the esterases [13]. Changes in enzymatic behaviour observed when lipases are employed in organic solvent systems have led to their use in the synthesis of lipids, carbohydrate esters, amines and amides, along with finding industrial application in the synthesis of pharmaceuticals, fine chemicals and cosmetics [14]. In these reactions, the lipases have been shown to exhibit excellent regio- and stereo-selectivity, enabling a facile route to the synthesis of optically active compounds [15–17].

**Scheme 1:**
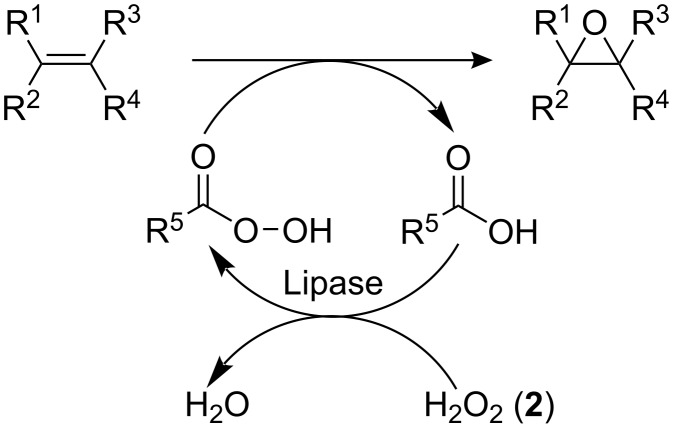
Illustration of the chemo-enzymatic epoxidation of an alkene; involving the biocatalytic perhydrolysis of a carboxylic acid followed by a peroxy acid epoxidation.

Chemo-enzymatic epoxidation provides an environmentally benign approach to the synthesis of epoxides, with Björkling and co-workers [18–19] initially reporting the generation of epoxides under mild conditions using seven lipases. As Scheme 1 illustrates, the biocatalytic epoxidation involves the lipase catalysed formation of a peroxy acid, from a carboxylic acid and an oxidant, such as H_2_O_2_ (**2**), which donates oxygen to the double bond, affording the respective epoxide or oxirane and regenerating the carboxylic acid.

Initial investigations illustrated the highest conversions in solvents such as hexane and toluene, with the lowest in dioxane and acetonitrile, following the lipase trend where the biocatalysts typically perform better in water immiscible solvents. Moderate deactivation of the enzyme was however observed, attributed to the use of high concentrations of H_2_O_2_ (**2**). This was overcome by adding aliquots of the oxidant over an extended period of time (4 h) affording the target epoxides in conversions ranging from 15% to quantitative depending on the alkene employed.

**Scheme 2:**
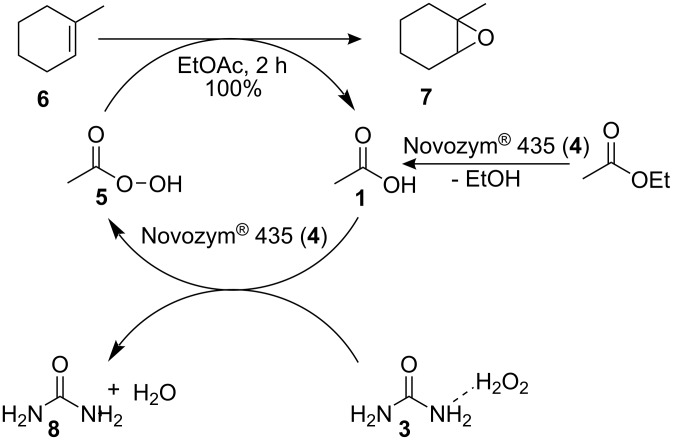
Illustration of the chemo-enzymatic epoxidation of 1-methylcyclohexene (**6**) to 1-methylcyclohexene oxide (**7**), using EtOAc as both a reactant and a solvent.

Törnvall et al. [20] subsequently conducted a detailed investigation into the source of enzyme deactivation, concluding that high temperature (60 °C) and high concentrations of H_2_O_2_ (**2**, 6 to 12 M) resulted in rapid loss of enzyme activity, again illustrating that careful dosage of H_2_O_2_ (**2**) increased the operational lifetime of the biocatalyst. The authors also noted that the presence of EtOH and urea resulted in moderate deactivation of the enzyme. In 2006, Olivo and co-workers [21] reported an environmentally benign method for alkene epoxidation *via* the chemo-enzymatic perhydrolysis of ethyl acetate using Novozym^®^ 435 (**4**), immobilised *Candida antarctica* lipase B, and UHP (**3**). They utilised the high specificity of *Candida antarctica* lipase B towards ester hydrolysis to generate acetic acid (**1**) in situ and subsequently synthesise the peracid **5** (Scheme 2). The authors found that compared to the use of aq H_2_O_2_ (**2**), the use of UHP (**3**) enabled controlled release of anhydrous H_2_O_2_ (**2**) and enabled the enzyme to be recycled with no observable effect on conversion over two catalytic cycles. Using this approach, Olivo et al. demonstrated the synthesis of twelve epoxides with yields ranging from 73% for 1-hexene to quantitative for 1-methylcyclohexene (**6**) and reaction times of 166 to 2 h respectively. Along with Björkling and co-workers [18–19], the authors found the use of aq H_2_O_2_ (**2**) resulted in a reduction in conversion after two cycles (60%) affording complete deactivation of the enzyme on the third cycle; yields in the range of 73 to 85% were however obtained upon adding the oxidant in aliquots over a period of 24 h.

Although the chemo-enzymatic epoxidation of alkenes is an attractive alternative to the hazardous reagents currently employed, finding use in the synthesis of the aroma linalool oxide and epoxidised soybean oil [16], the reaction times employed for the transformations (< 166 h) and observed reductions in enzyme activity in the presence of H_2_O_2_ (**2**) do not currently make the technique a practical alternative to traditional techniques. With this in mind, we report herein the development and evaluation of continuous flow technique for the enzyme-mediated synthesis of epoxides, employing the immobilised *Candida antarctica* lipase B, Novozym^®^ 435 (**4**). This was recently found to be the most efficient biocatalyst with respect to peracid formation, facilitating a series of oxidations including alkenes [21] and ketones *via* the Baeyer–Villiger reaction [22] and retaining the broad substrate specificity of *Candida antarctica* lipase B.

In the past decade micro reactors, and more generically continuous flow reactors, have been shown to offer many advantages to the synthetic chemist, such as reduced reactions times, increased catalytic efficiency, increased product purity and atom efficiency [23–26]. More recently, authors have begun to incorporate biocatalysts into flow reactors as a means of increasing productivity, biocatalyst lifetimes and exploring the potential of employing immobilised enzymes in industrial processes. Recent examples include the continuous flow enantioselective acetylation of a series of racemic secondary alcohols [27] and the continuous flow synthesis of alkyl esters [28].

## Results and Discussion

Combining our experience of micro reaction methodology with the observations made by Olivo and co-workers [21–22], it was proposed that by conducting the lipase-mediated epoxidation under continuous flow it would be possible to reduce the reaction times required to oxidise synthetically useful alkenes, whilst increasing the biocatalyst’s lifetime. With this in mind, our initial investigation into the development of a continuous process for the synthesis of epoxides focussed on the incorporation of immobilised *Candida antarctica* lipase B, Novozym^®^ 435 (**4**), into a flow reactor and the use of urea–hydrogen peroxide (**3**) as the oxidising agent.

**Scheme 3:**
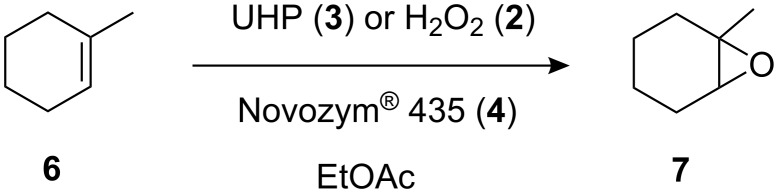
Model reaction used to compare the continuous flow epoxidation strategy with the conventional batch technique.

**Figure 1:**
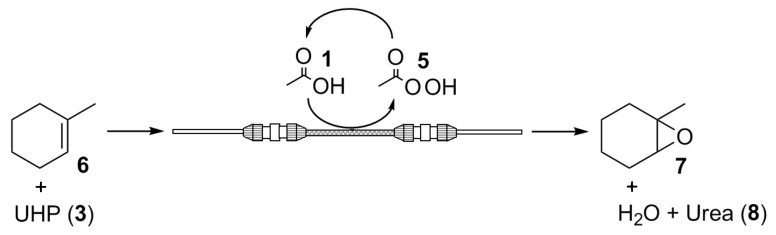
Schematic of the reaction set-up used to evaluate the continuous flow chemo-enzymatic epoxidation of 1-methylcyclohexene (**6**) *via* the perhydrolysis of acetic acid (**1**).

In addition to reports made by Olivo and co-workers, UHP (**3**) [29] was selected as the oxidant as it is a cheap, commercially available, source of anhydrous H_2_O_2_ (**2**) which, in addition to its use in the synthesis of epoxides [30–31], has found widespread application in the conversion of nitriles to amides [32], aldehydes to acids [33], and sulfides to sulfones [34]. To enable comparison of the method developed here with batch investigations previously conducted, the oxidation of 1-methylcyclohexene (**6**) to 1-methylcyclohexene oxide (**7**) was selected as a model reaction (Scheme 3) for investigation within the reaction set-up illustrated in Figure 1.

### Micro reactor set-up

As Figure 1 depicts, the flow reactor consisted of a borosilicate glass capillary (3.0 mm (i.d.) × 3.6 cm (long) packed with 100 mg of Novozym^®^ 435 (**4**), with reactions conducted by pumping a pre-mixed solution of the alkene **6** and UHP (**3**) through the packed-bed using a syringe pump.

The reaction products were collected in a sample vial containing EtOAc (1 ml) over a period of 10 min, and analysed by GC-MS in order to quantify the conversion of 1-methylcyclohexene (**6**) to 1-methylcyclohexene oxide (**7**). Using this approach enabled rapid screening of the reaction conditions to be conducted: if residual alkene **6** was detected the reaction was simply repeated employing an increased reaction time, which was achieved by reducing the flow rate of the reactant stream. Upon changing the reaction conditions, the system was allowed to equilibrate for 30 min prior to sampling, thus ensuring equilibration of the reactor. In addition, to ensure that meaningful data was obtained, all reaction conditions were monitored for a minimum of 5 samples (*n*) and the average result reported along with the relative standard deviation (% RSD).

### Evaluation of UHP as an oxidant

When adapting or developing reaction methodology for a continuous flow application, it is imperative to ensure that all reactants, by-products and products remain soluble over the course of the reaction, so as to prevent blockage formation within the fluidic system which can lead to the build-up of pressure followed by leaking from any interconnections. Consequently, due to the limited solubility of UHP (**3**) in those organic solvents found to promote the epoxidation of alkenes (typically ethyl acetate and DCM) the use of conditions analogous to those reported within the literature (~0.3 M) were not feasible within the flow reactor. As such, an ethyl acetate solution of reduced concentration [alkene **6** (2.5 × 10^−2^ M) and UHP (**3**) (5.0 × 10^−2^ M)] was employed and initial investigations focussed on evaluating the effect of reaction time on the epoxidation of 1-methylcyclohexene (**6**). Employing a flow rate of 10 μl min^−1^, hence a residence time of 2.6 min, it was gratifying to observe that under continuous flow conditions, enzymatic hydrolysis of ethyl acetate to acetic acid (**1**) followed by formation of peracetic acid (**5**) and finally oxidation of 1-methylcyclohexene (**6**) had occurred, albeit in low conversion (5.1%). Varying the flow rate of the reactant solution through the packed bed between 0.1 and 50 μl min^−1^, at 27 °C, enabled us to identify 0.1 μl min^−1^ (4.3 h residence time) as the optimum flow rate for the synthesis of 1-methylcyclohexene oxide (**7**, 89.0%).

Using such a low flow rate is however impractical as it would only generate 6.0 × 10^−3^ mg h^−1^ of 1-methylcyclohexene oxide (**7**), thus providing no practical advantage over a conventional, stirred batch reaction. Although a facile approach to increasing the throughput of flow reactions is to increase the concentration of the reactant stream, in this case the limited solubility of UHP (**3**) precludes this as a viable option.

### H_2_O_2_ as an alternative oxidant

As a means of increasing the reactor throughput, an alternative oxidant was sought. H_2_O_2_ (**2**) was initially considered as it presented the distinct advantage over UHP (**3**) that it is soluble in the selected reaction solvent, ethyl acetate, enabling it to be employed at a higher concentration if required. Although batch investigations had highlighted problems associated with the use of H_2_O_2_ (**2**) in the chemoenzymatic epoxidation of alkenes, with it found to deactivate *Candida antarctica* lipase B [35] and prevent recycling of the biocatalyst, it was proposed that by conducting the reactions under continuous flow, where at any one time the quantity of oxidant **2** in contact with the enzyme would be low, continued operation and efficient recycling would be feasible.

**Figure 2:**
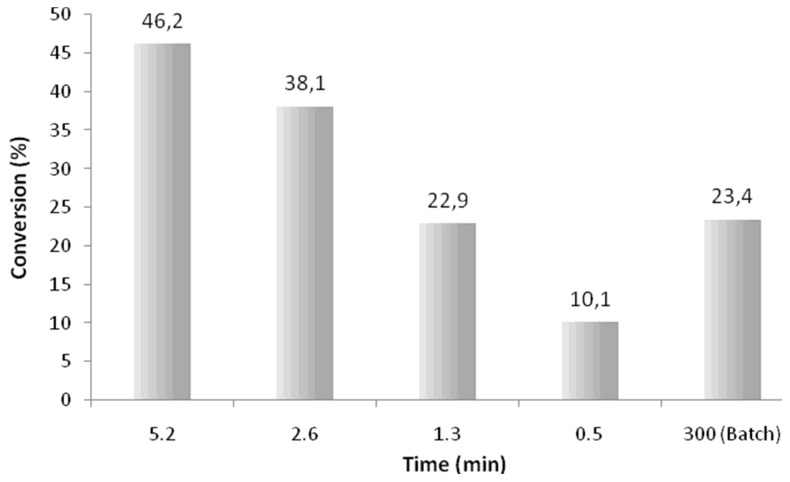
Graph illustrating the effect of flow rate (hence residence time) on the conversion of 1-methylcyclohexene (**6**) to 1-methylcyclohexene oxide (**7**) at room temperature (27 °C); employing 2.0 equiv of H_2_O_2_ (**2**).

As the aim of the investigation was to develop a continuous flow process for the synthesis of epoxides in high purity, it was decided at this stage to employ 5 μl min^−1^ as the minimum flow rate and increase the reactant concentrations by a factor of four; the 2:1 oxidant to alkene ratio was however maintained. With these factors in mind, flow reactions were performed by pumping a pre-mixed solution of 1-methylcyclohexene (**6**, 0.1 M) and H_2_O_2_ (**2**, 0.2 M), in ethyl acetate, through the reactor (0.10 g Novozym^®^ 435 (**4**)) at a flow rate of 50 μl min^−1^ (0.52 min residence time). After a short equilibration time, 30 min, the reaction products were once again collected at ten minute intervals and analysed by GC-MS in order to determine the percentage conversion of the alkene **6** to 1-methylcyclohexene oxide (**7**). As Figure 2 illustrates, at room temperature a moderate conversion of 10.1% was obtained, this was however improved upon by systematically reducing the flow rate, which had the effect of increasing the residence time from 0.52 to 5.20 min and afforded an optimal conversion of 46.2% at a flow rate of 5 μl min^−1^ (5.20 min).

To ensure that the results obtained were as a result of employing a packed-bed reactor and to exclude the possibility that the reaction was continuing within the collection vial, as only the hydrolysis and perhydrolysis steps were enzyme catalysed, five samples were analysed immediately and then subjected to re-analysis 5 h later. Analogous results were obtained within experimental error (± 0.1%), confirming that the reaction occurred only within the packed bed.

Although up to this point we had failed to oxidise the alkene **6** completely, the use of H_2_O_2_ (**2**) was found to be advantageous compared to UHP (**3**) as it removed the issues associated with reagent insolubility and enabled flow reactions to be conducted at increased alkene concentrations, with product isolation simplified as the only by-product from H_2_O_2_ (**2**) is water. In addition, compared to an analogous batch reaction, it can be seen that equivalent results can be obtained within the flow reactor with reaction time of 1.3 min (22.9%) compared to 5 h (23.4%) in batch and improved conversions attainable with a reaction time of 5.2 min (46.2%). This approach is therefore advantageous as it provides a facile means of continuously adding the oxidant **2**, therefore removing the need for the time consuming practise of adding aliquots of the oxidant **2** in order to maintain the enzymes activity.

### Effect of reaction temperature on the oxidation reaction

Novozym^®^ 435 (**4**) has been shown to retain activity when heated to 60 °C, however in the presence of H_2_O_2_ (**2**) the enzyme has been reported to lose activity, with a half life of 3.9 h at 60 °C [6.0 M H_2_O_2_ (**2**)] [20]. It was therefore proposed that due to the short contact times between the enzyme and oxidant **2** within the flow reactor, denaturation of the enzyme by the oxidant **2** would be avoided; consequently the effect of reactor temperature on the epoxidation of 1-methylcyclohexene (**6**) was investigated.

When heating a flow reactor, it is important to consider the boiling point of the reactants and solvent system as any changes in viscosity, and even boiling, of the components can lead to irreproducible residence times within the reactor; at 5 μl min^−1^ and 60 °C, 64.5 ± 14.5% (*n* = 5) 1-methylcyclohexene oxide (**7**) was obtained. To ensure that a uniform residence time was obtained, a back-pressure regulator (100 psi) was inserted into the reaction set-up (between the packed-bed and the collection tube), enabling heating of the reactor above the boiling point of the solvent − in this case 77 °C. To supply heat to the bioreactor, a commercially available HPLC column heater was employed and the effect of reaction temperature on the rate of epoxidation assessed (40 to 70 °C) over a range of flow rates. Incorporation of a BPR into the reaction system reduces the error associated with the process (± 0.1%) and is a facile means of obtaining stable, plug flow at elevated reaction temperatures.

**Figure 3:**
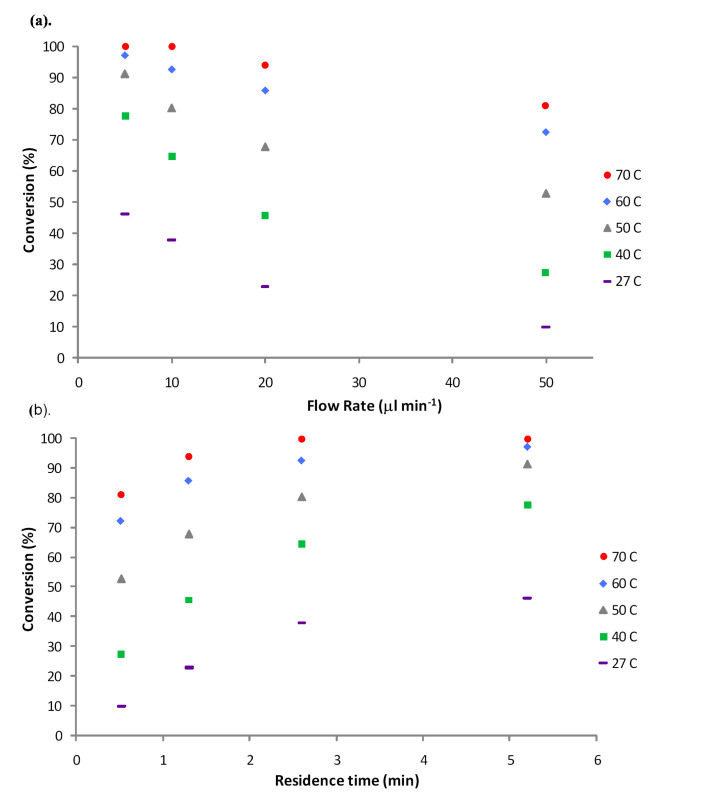
Graph illustrating the effect of (a) flow rate and (b) residence time on the conversion of 1-methylcyclohexene (**6**) to 1-methylcyclohexene oxide (**7**) at various reaction temperatures (27 to 70 °C).

Although the percentage of epoxide **7** synthesised was found to increase with reducing flow rate, when comparing the data obtained at room temperature with that collected at elevated temperatures (40 and 70 °C), it can be seen that the oxidation of 1-methylcyclohexene (**6**) increased most notably with reaction temperature; whereby quantitative conversion was obtained at 70 °C, cf. 38.1% **7** at 27 °C (10 μl min^−1^) (Figure 3). Under the optimised conditions stated, 1-methylcyclohexene oxide (**7**) was synthesised at a throughput of 6.7 mg h^−1^ from a single reactor with post reactor processing consisting simply of an aqueous extraction to remove water and acetic acid (**1**) from the epoxide **7**.

Having subjected the same portion of enzyme **4** to a prolonged thermal regime (21 h), it was imperative to ensure that the enzyme had not lost activity; to evaluate this a flow reaction was performed using the initial reaction conditions of 5 μl min^−1^ at room temperature. Using this approach, 46.3% 1-methylcyclohexene oxide (**7**) was obtained, which compared favourably to the 46.2% **7** obtained prior to heating the bioreactor; confirming enzyme activity was maintained.

### Effect of oxidant concentration on enzyme stability

**Figure 4:**
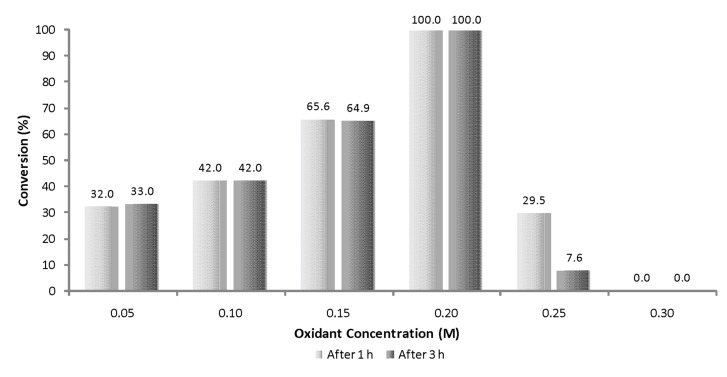
Graph illustrating the effect of oxidant stoichiometry on the conversion of 1-methylcyclohexene (**6**) to 1-methylcyclohexene oxide (**7**) at a residence time of 2.6 min (70 °C).

Although it was pleasing to observe stability of the Novozym^®^ 435 (**4**) at various reaction temperatures, with literature precedent reporting enzyme deactivation at an oxidant concentration of 5.0 × 10^−2^ M the next step of the investigation was aimed at evaluating the enzyme’s tolerance to H_2_O_2_ (**2**) in the micro reactor. To achieve this, the concentration of alkene **6** was maintained at 0.1 M and the oxidant **2** concentration varied between 5 × 10^−2^ and 0.3 M (0.5 to 3.0 equiv with respect to alkene **6**) and the reactions conducted at 10 μl min^−1^ (2.6 min) and 70 °C, with samples taken after 1 and 3 h of continuous operation. As Figure 4 illustrates, when employing 0.5 equiv of oxidant **2** an average of 32.5% conversion was obtained. This rose to 42.0% when employing equimolar quantities of H_2_O_2_ (**2**), whereas employing a slight excess afforded 65.3% conversion (1.5 equiv) and 2 equiv again affording quantitative conversion to 1-methylcyclohexene oxide (**7**). Interestingly when a concentration of 2.5 M H_2_O_2_ (**2**) was employed, rapid deactivation of the enzyme was observed, illustrated by a reduction in conversion to 29.5% after 1 h and 7.6% after 3 h. To confirm that the enzyme had undergone irreversible damage, the reactor was purged with ethyl acetate (2.5 ml) and the reaction utilising 2 equiv of H_2_O_2_ (**2**) repeated at 70 °C, whereby baseline formation of the epoxide was observed. Prior to conducting investigations at 0.3 M, the Novozym^®^ 435 (**4**) was removed and a second portion of the biocatalyst packed into the reactor (0.10 g), again however deactivation was observed, this time after only 1 h. Although deactivation of the enzyme was observed at 2.5 and 3.0 M H_2_O_2_ (**2**), the biocatalyst **4** was found to be tolerant to four times the oxidant concentration (0.2 M) compared to stirred and shaken batch investigations (0.05 M), illustrating another advantage associated with micro reaction technology.

### Monitoring enzyme stability over an extended period of operation

Having confirmed that the optimum conditions for the synthesis of 1-methylcyclohexene oxide (**7**) were a residence time of 2.6 min, a reactor temperature of 70 °C and an oxidant **2**/alkene **6** ratio of 2:1, the stability of the reaction system was monitored over a period of 24 h using fresh biocatalyst (0.10 g **4**). In order to highlight any fluctuations in the system, along with demonstrating continuous operation of analytically pure **7** under the optimised conditions, the system was also operated at the sub-optimal temperature of 27 °C. Samples were collected every 10 min for 7.5 h, then once an hour for 9 h, after an 8 h break in sampling; for visual clarity, over the initial 7.5 h every other data point is plotted.

**Figure 5:**
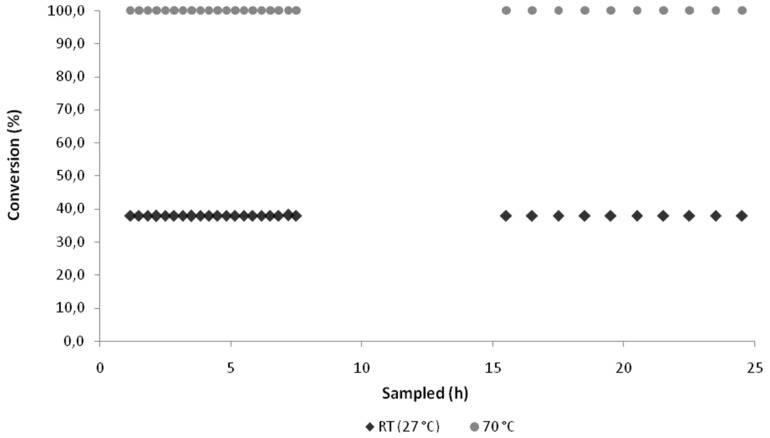
Illustration of the enzyme **4** stability to H_2_O_2_ (**2**) for the conversion of 1-methylcyclohexene (**6**) to 1-methylcyclohexene oxide (**7**) (residence time = 2.6 min) at 27 °C and 70 °C over a 24 h period.

As Figure 5 illustrates, in both cases stable operation was observed over the 24 h period, with no fluctuations in the purity of the epoxide **7** synthesised at 70 °C and 0.08% RSD observed for the reaction conducted at room temperature.

### Effect of substrate

Having demonstrated the ability to rapidly optimise the reaction conditions required for the chemo-enzymatic epoxidation of 1-methylcyclohexane (**6**), attaining quantitative conversion to **7** with a reactor temperature of 70 °C and a reaction time of 2.6 min, the investigation was extended to evaluate the generality of the methodology developed. Employing the aforementioned conditions, the epoxidation of a series of alkenes was conducted and the results obtained are summarised in Table 1.

**Table 1:** Summary of the reaction conditions employed for the lipase-mediated epoxidation of an array of alkenes under continuous flow.

Alkene	Temperature (°C)	Residence Time (min)^b^	Conversion (%)	Yield (%)^b^

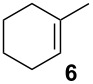	70	2.6 (2)	100.0	99.1 (6.7)^c^
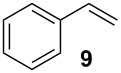	70 70	2.6 5.2 (33)	57.2 100.0	– 99.2 (3.6)
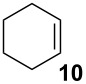	70	2.6 (40)	100.0	97.6 (5.9)
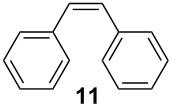	70 70	2.6 5.2	31.9 100.0	– 99.5 (5.9)
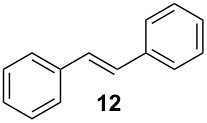	70 70	2.6 5.2	32.1 100.0	– 99.1 (5.9)

The number in parentheses represents ^a^the reaction time required under batch conditions (h) [21], ^b^the isolated yield obtained after continuous operation of the reactor for 24 h and ^c^the throughput (mg h^−1^) using the optimised conditions.

**Scheme 4:**
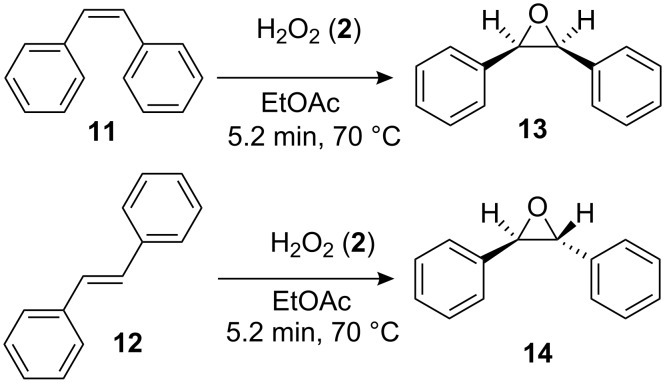
Illustration of the reaction products obtained when conducting the continuous flow epoxidation of *cis*-stilbene (**11**) and trans-stilbene (**12**).

In the case of styrene (**9**), incomplete conversion was obtained with a residence time of 2.6 min hence the flow rate was reduced to 5 μl min^−1^ (5.2 min) whereby quantitative conversion to styrene oxide was obtained; continuous operation of the reactor, followed by an aqueous extraction, afforded the target compound in 99.2% isolate yield. Cyclohexene (**10**) was readily converted to cyclohexene-1,2-oxide at 70 °C, 2.6 min residence time, and was isolated in 97.6% yield. In addition to evaluation of aromatic and aliphatic alkenes discussed, the epoxidation of *cis*-stilbene (**11**) and *trans*-stilbene (**12**) was investigated (Scheme 4).

Due to the molecule’s symmetry, the configuration of the epoxides synthesised could not be characterised by ^1^H NMR spectroscopy; however the compounds did possess significantly different retention times (*t*_R_) when analysed by GC-MS; *cis*-1,2-diphenyloxirane (**13**, *t*_R_ = 9.81 min) and *trans*-1,2-diphenyloxirane (**14**, *t*_R_ = 10.28). Consequently, GC-MS in conjunction with a synthetic standard of *trans*-1,2-diphenyloxirane (**14**) was used to confirm the geometric isomer synthesised. In both cases, the optimal reaction conditions were 70 °C and a residence time of 5.2 min, affording the *cis*-isomer **13** in 99.5% yield as colourless oil and the *trans*-isomer **14** in 99.1% yield as a white crystalline solid. In all cases, the epoxides were synthesised in excellent yield and purity with no hydrolysis products observed.

In comparison to the reaction times employed in analogous batch reactions, Table 1, the examples reported herein serve to illustrate the dramatic reductions in reaction time attainable through the use of micro reaction technology; in the case of the epoxidation of styrene (**9**), a 381-fold reduction in reaction time is obtained by employing H_2_O_2_ (**2**) under continuous flow. In addition to the reduced reaction times, the continuous flow methodology also enables larger quantities of oxidant **2** to be used, compared to conventional batch systems where deactivation of *Candida antarctica* lipase B was observed with 25% less oxidant **2**. Consequently, the oxidation of styrene (**9**) can be performed at a throughput of 3.6 mg h^−1^ compared to 0.1 mg h^−1^ in batch. Furthermore, the continuous reactor capacity can be increased by employing additional packed-bed reactors, operated under identical reaction conditions, compared to batch where increases in reactor volume/capacity will result in the need for longer dosing times for the oxidant **2**.

## Conclusion

In conclusion, we have demonstrated the successful transfer of a chemo-enzymatic batch process to a packed-bed flow reactor, enabling a dramatic reduction in reaction time to be obtained. In addition, the use of a flow reactor enabled higher quantities of H_2_O_2_ (**2**) to be employed over prolonged periods of time (> 24 h) without deactivation of the biocatalyst. Using this approach has enabled the development of a robust and operationally simple technique for the chemo-enzymatic epoxidation of olefins.

In the examples reported herein, we focussed on the use of ethyl acetate as it acted as both a solvent and reagent with in situ hydrolysis providing a source of acetic acid (**1**), from which the peracid **5** could be generated. It is however acknowledged that other solvents may be required and future work will therefore focus on the use of alternative solvents systems, employing a catalytic quantity of a carboxylic acid additive to generate the respective peracid, enabling greater system flexibility, along with evaluating the methodology towards other synthetically useful oxidations.

## Experimental

### Materials

1-Methylcyclohexene (**6**, 99%, Aldrich), styrene (**9**, ReagentPlus^®^, ≥ 99%, Aldrich), *cis*-stilbene (**11**, 96%, Aldrich), *trans*-stilbene (**12**, 96%, Aldrich), cyclohexene (**10**, 99%, Aldrich), urea–hydrogen peroxide (**3**, Aldrich), hydrogen peroxide (**2**, 30%, 100 volume, Fisher Scientific), Novozym^®^ 435 (**4**) (Lipase acrylic resin from *Candida antarctica*, ≥ 10 000 U g^−1^, Nordisk), deuterated chloroform (+0.03% TMS, < 0.01% H_2_O, Euriso-top) and ethyl acetate (Reagent grade, Fisher Scientific). In all cases, materials were used as received.

### Instrumentation

Unless otherwise stated, nuclear magnetic resonance (NMR) spectra were obtained at room temperature as solutions in deuterated chloroform (CDCl_3_) using a Jeol GX400 spectrometer; in the case of known compounds, all spectra obtained were consistent with the literature. The following abbreviations are used to report NMR spectroscopic data; s = singlet, d = doublet, t = triplet, br s = broad singlet, q = quartet, dd = double doublet, dt = doublet of triplets, m = multiplet and C_0_ = quaternary carbon. Analysis of samples by Gas Chromatography-Mass Spectrometry (GC-MS) were performed using a Varian GC (CP-3800) coupled to a Varian MS (2100T) with a CP-Sil 8 (30 m) column (Phenomenex, UK) and ultra high purity helium (99.9999%, Energas, UK) as the carrier gas. Reactions employing 1-methylcyclohexene (**6**), cyclohexene (**10**), styrene (**9**), *cis*-stilbene (**11**) and *trans*-stilbene (**12**) were analysed using the following method; injector temperature 200 °C, carrier gas flow rate 1.00 ml min^−1^, oven temperature 50 °C for 4 min then ramped to 150 °C at 30 °C min^−1^ and held at 150 °C for 6.17 min, with a 2.5 min filament delay. Mass spectrometry data was obtained using a Shimadzu QP5050A instrument with an EI ionisation source. Batch reactions were conducted using a carousel reactor/rotator (SB2, Stuart) to reduce mechanical degradation of the immobilised enzyme. Flow reactions were conducted using a displacement pump (MD-1001, Bioanalytical Systems Inc.), capable of delivering three solutions at flow rates between 0.1 and 100 μl min^−1^ (calibrated for a 1 ml gas-tight syringe) and solutions delivered to the flow reactor using a 2.5, 10 or 20 ml gas-tight syringe (Hamilton, UK). The packed-bed consisted of a borosilicate glass column (3.0 mm (i.d.) × 3.6 cm (long)) (Omnifit), the immobilised enzyme was held in place using 25 μm polyethylene frits and fluidic connections made using commercially available PEEK and PTFE connectors (Supelco). The flow reactor was heated using a HPLC programmable column heater, containing stainless steel blocks (Jones Chromatography) and the reactor temperature monitored using a thermocouple (206-3750, RS). When heating the reactor in excess of 50 °C, reactions were conducted under pressure, using a back-pressure regulator cartridge (100 psi) housed within a stainless steel holder (Upchurch Scientific, IDEX Corporation), to prevent boiling of the reactants and solvent system.

### General procedure for the epoxidation of olefins in batch

To a solution of Novozym^®^ 435 (**4**, 0.10 g) and UHP (**3**, 1.0 mmol) in ethyl acetate (5 ml), was added the alkene (0.5 mmol) and the reaction mixture shaken on a carousel reactor at room temperature. Upon completion of the reaction, as indicated by TLC, the reaction mixture was filtered through a plug of cotton wool and the organic solvent evaporated prior to dissolution of the residue in DCM (20 ml). The organic layer was then washed with an aq solution of saturated NaHCO_3_ (2 × 20 ml) to remove any acetic acid (**1**) and residual urea (**8**). The organic layer was then dried using MgSO_4_, filtered and concentrated *in vacuo* to afford the target epoxide. The reaction products were then analysed by GC-MS, ^1^H and ^13^C NMR spectroscopy and the spectroscopic data compared with those reported within the literature.

### General procedure for the epoxidation of olefins in batch using H_2_O_2_ (**2**)

To a stirred solution of Novozym^®^ 435 (**4**, 0.10 g) and alkene (0.5 mmol) in ethyl acetate (5 ml) was added H_2_O_2_ (**2**, 1.0 mmol) in aliquots of 0.1 mmol 15 min^−1^, dispensed from an autopipettor. Upon completion of the reaction, as indicated by TLC, the reaction mixture was filtered through a plug of cotton wool and the organic solvent evaporated prior to dissolution of the residue in DCM (20 ml). The organic layer was then dried using MgSO_4_, filtered and concentrated *in vacuo* to afford the target epoxide. The reaction products were then analysed by GC-MS, ^1^H and ^13^C NMR spectroscopy and the spectroscopic data compared with those reported within the literature.

### General procedure for the optimisation of olefin epoxidation under continuous flow

To optimise a continuous flow epoxidation, a gas-tight syringe (2.5 ml) was filled with a solution containing the alkene under investigation (0.1 M), oxidant (0.2 M) in ethyl acetate and connected to the Omnifit reactor cartridge using PTFE tubing [150 μm (i.d.) × 10 cm (long)] and a PEEK luer connector coupled to a 1/16″ HPLC connector. To the pre-weighed reactor was added Novozym^®^ 435 (**4**, 0.10 g, 26 μl total volume) and the reactor outlet formed from a length of PTFE tubing [150 μm (i.d.) × 5 cm (long)] to enable efficient sampling. The reaction mixture was then pumped through the packed bed (5 to 50 μl min^−1^) for a period of 30 min, in order to equilibrate, prior to collection of the reaction products every 10 min for a minimum of 50 min. The resulting reaction products were analysed by GC-MS in order to determine the conversion of alkene to epoxide. If residual alkene was detected, the reaction was repeated at a slower flow rate to increase the reaction time (0.52 to 5.2 min).

To increase the rate of epoxidation, reactions were also conducted at elevated reaction temperatures (27 to 70 °C), with heating achieved using a stainless steel column heater and the reaction temperature monitored using a thermocouple. When employing reaction temperatures > 50 °C, a back-pressure regulator (100 psi) was incorporated into the system at the reactor outlet in order to prevent boiling of the reactants, products and solvent. Samples were again taken at intervals of 10 min and analysed off-line by GC-MS to determine the percentage of epoxide synthesised.

### General protocol used to synthesise epoxides under continuous flow

In order to deliver a constant stream of reactants through the packed-bed, a 20 ml gas-tight syringe (10 ml) was filled with a pre-mixed solution containing the alkene under investigation (0.1 M) and 30% hydrogen peroxide (**2**, 0.2 M) in ethyl acetate. The reaction mixture was then pumped through the packed-bed, containing Novozym^®^ 435 (**4**, 0.10 g, 26 μl total volume), under the previously optimised reaction conditions for 12 h. The reaction mixture was then concentrated in vacuo to remove the reaction solvent, prior to dissolution of the residue in DCM (20 ml). The organic layer was then washed with an aq solution of saturated NaHCO_3_ (2 × 20 ml) to remove any acetic acid and residual peracetic acid (**5**). The organic layer was then dried using MgSO_4_, filtered under suction and the filtrate concentrated *in vacuo* to afford the target epoxide. The reaction product was then analysed by GC-MS, ^1^H and ^13^C NMR spectroscopy in order to characterise the epoxide and determine product purity.

### Enzyme stability

To confirm the Novozym^®^ 435 (**4**) was sufficiently stable to be used for the continuous flow synthesis of epoxides over extended periods of operation, two experiments were conducted over a period of 24 h. In both cases a 20 ml solution of 1-methylcyclohexene (**6**, 0.1 M) and hydrogen peroxide (**2**, 0.2 M) in ethyl acetate was pumped through the reactor at a flow rate of 10 μl min^−1^, with one reaction performed at 27 °C and the other at 70 °C. Samples were taken every 10 min for 7.5 h and then every 1 h for 9 h after a break in sampling of 8 h; operation was maintained for these 8 h but no samples taken. At 27 °C, an average conversion to 1-methylcyclohexene oxide (**7**) of 37.9% (% RSD = 0.08) was obtained and at 70 °C, quantitative conversion was obtained over the 24 h period.

### 1-Methylcyclohexene oxide (**7**)

A pre-mixed solution of 1-methylcyclohexene (**6** ,0.1 M) and 30% H_2_O_2_ (**2**, 0.2 M) was pumped through the enzyme filled reactor, at a flow rate of 10 μl min^−1^ and a reaction temperature of 70 °C, for a period of 24 h to afford 1-methylcyclohexene oxide (**7**) as a colourless oil (0.160 g, 99.1% yield) after an aqueous extraction; ^1^H NMR (400 MHz, CDCl_3_) δ 1.49–1.68 (m, 4H), 1.65 (s, 3H), 1.89–2.20 (m, 4H) and 2.91 (m, 1H); ^13^C NMR (100 MHz, CDCl_3_) δ 17.6 (CH_3_), 19.9 (CH_2_), 20.1 (CH_2_), 25.2 (CH_2_), 26.1 (CH_2_), 52.1 (CH) and 62.2 (C_0_); *m/z* (EI) 113 (M^+^+1, 20%), 112 (10), 97 (77), 85 (15), 95 (100), 83 (15), 69 (15) and 55 (25); GC-MS *t*_R_ = 5.26 min. The spectroscopic data obtained for 1-methylcyclohexene oxide (**7**) was consistent with that reported within the literature [36].

### 1,2-Epoxy-1-phenylethane (styrene oxide)

A pre-mixed solution containing styrene (**9**, 0.1 M) and 30% H_2_O_2_ (**2**, 0.2 M) in ethyl acetate was pumped through the enzyme filled reactor at a flow rate of 5 μl min^−1^ and a reaction temperature of 70 °C for a period of 24 h. The resulting reaction products were concentrated *in vacuo* and the residue subjected to an aqueous extraction, affording 1,2-epoxy-1-phenylethane as a colourless oil (0.086 g, 99.2% yield); ^1^H NMR (400 MHz, CDCl_3_) δ 2.75 (dd, *J* = 2.8, 5.6, 1H), 3.08 (dd, *J* = 4.1, 5.6, 1H), 3.80 (dd, *J* = 2.8, 4.1, 1H) and 7.24–7.33 (m, 5H); ^13^C NMR (100 MHz, CDCl_3_) δ 51.0 (CH_2_), 51.1 (CH), 125.3 (2 × CH), 128.0 (CH), 128.2 (2 × CH) and 137.5 (C); *m/z* (EI) 121 (M^+^+1, 5%), 120 (20), 119 (50), 92 (25), 91 (100), 89 (40) and 77 (20); GC-MS *t*_R_ = 5.37 min. The spectroscopic data obtained for 1,2-epoxy-1-phenylethane was consistent with that reported within the literature [37].

### *cis*-1,2-Diphenyloxirane (*cis*-stilbene oxide, **13**)

A solution containing *cis*-stilbene (**11**, 0.1 M) and 30% H_2_O_2_ (**2**, 0.2 M) in ethyl acetate was pumped through the packed-bed reactor (70 °C) at a flow rate of 5 μl min^−1^ and the reactor effluent collected over a period of 24 h. The reaction products were concentrated *in vacuo* and subjected to an aqueous extraction to afford *cis*-stilbene oxide (**13**) as a colourless oil (0.140 g, 99.5% yield); ^1^H NMR (400 MHz, CDCl_3_) δ 4.31 (s, 2H), 7.09–7.21 (m, 10H); ^13^C NMR (100 MHz, CDCl_3_) δ 59.9 (2 × CH), 125.9 (4 × CH), 127.2 (2 × CH), 127.5 (4 × CH) and 135.6 (2 × C); *m/z* (EI) 197 (M^+^+1, 100%), 196 (75), 195 (76), 167 (75), 152 (10), 105 (50), 89 (25) and 77 (10); GC-MS *t*_R_ = 9.81 min. The spectroscopic data obtained for *cis*-1,2-diphenyloxirane (**13**) was consistent with that reported within the literature [38].

### *trans*-1,2-Diphenyloxirane (*trans*-stilbene oxide, **14**)

A pre-mixed solution of *trans*-stilbene (**12**, 0.1 M) and 30% H_2_O_2_ (**2**, 0.2 M) was pumped through the enzyme filled reactor, at a flow rate of 5 μl min^−1^ and a reaction temperature of 70 °C, for a period of 24 h to afford *trans*-stilbene oxide (**14**) as a white crystalline solid (0.140 g, 99.5% yield); ^1^H NMR (400 MHz, CDCl_3_) δ 3.87 (s, 2H) and 7.25–7.59 (m, 10H); ^13^C NMR (100 MHz, CDCl_3_) δ 62.8 (CH), 125.5 (4 × CH), 128.3 (2 × CH), 128.5 (4 × CH) and 137.1 (2 × C); *m/z* (EI) 197 (M^+^+1, 70%), 196 (75), 195 (73), 180 (65), 167 (100), 165 (50), 105 (25), 89 (20) and 77 (10); GC-MS *t*_R_ = 10.28 min. The spectroscopic data obtained for *trans*-1,2-diphenyloxirane (**14**) was consistent with that reported within the literature [39].

### 1,2-Epoxycyclohexane (cyclohexene oxide)

A pre-mixed solution of cyclohexene (**10**, 0.1 M) and 30% H_2_O_2_ (**2**, 0.2 M) was pumped through the packed-bed reactor at a flow rate of 5 μl min^−1^ and a reaction temperature of 70 °C for a period of 24 h. The reaction products were concentrated *in vacuo* and the residue subjected to an aqueous work up, affording 1,2-epoxycyclohexane as a colourless oil (0.138 g, 97.6% yield); ^1^H NMR (400 MHz, CDCl_3_) δ 1.15–1.27 (m, 2H), 1.31–1.49 (m, 2H), 1.65–1.99 (m, 4H) and 3.11 (s, 2H); ^13^C NMR (100 MHz, CDCl_3_) δ 19.9 (2 × CH_2_), 25.1 (2 × CH_2_) and 52.2 (2 × CH); *m/z* (EI) 99 (M^+^+1, 5%), 98 (7), 97 (20), 83 (100), 81 (80), 69 (20) and 55 (25); GC-MS *t*_R_ = 4.876 min. The spectroscopic data obtained for 1,2-epoxycyclohexane was consistent with that reported within the literature [40].
